# Unhealthy Localities

**Published:** 1876-08

**Authors:** 


### UNHEALTHY LOCALITIES.

All things considered, it is very questionable whether location has much to do with the health of individuals. We are accustomed to say that mountainous districts must be healthy because we find there pure air and water, and all the conditions essential to health and long life. Still, the vital statistics of such places prove that the average term of human existence is less than among the inhabitants of level countries.

Now, it is evident that the predisposing causes of disease exist, to a great extent, in low countries, and it seems anomalous that more persons will attain long life there than at places far more salubrious.

But it is easily explained. "Self-preservation is the first law of our nature." It is this that causes us intuitively to protect ourselves against outward enemies, whether wild beasts or insidious diseases.

"To be forewarned is to be forearmed." If, in any district, fatigue and exposure in the night air, and the air of the early morning, are pretty certain to be followed by "chills and fever," we know that malaria exists in the air, and we protect ourselves and our families against its influence. Thus we guard ourselves against all enemies, both animate and inanimate.

If we are sensible people, we surround ourselves with as many home comforts as possible, and endeavor to enjoy them during those hours when the thoughtless and heedless are exposing themselves to fever. We lead quiet lives, are regular in our habits, possess our souls in peace, and thus disarm all enemies and attain a vigorous old age. The inhabitants of mountainous districts frequently err in the direction of over-confidence in the salubrity of their climate. We are acquainted with hundreds of men who have been made invalids for life by fool-hardy exposure to the chilly night air of the mountains. Most of them considered themselves proof against sudden changes in temperature, and did not think it necessary to provide against them.

Such cases increase the death rate of regions which should be the most healthy, and make the low lands seem the most favorable to long life. Wherever we reside it is a duty to care for our health ; and it should never be considered a task. In malarious districts it is not well to leave the house in the morning to perform any outdoor labor until an hour or two after sunrise, and after a substantial breakfast. It is not safe to sit down out of doors and remain quiet for any considerable time in the early evening. The air of the early morning and evening is the most unhealthy. Since "an ounce of prevention is worth a pound of cure," we advise that from two to ten grains of quinine should be taken in a tablespoonful of strong, cold coffee, whenever there is a sense of weariness and aching of the head and back, and a feeling of general discomfort. These are the premonitory symptoms of "intermittent fever," and this is the time to employ our remedies without waiting till the disease fastens itself for a three-months' siege.

It is easier to keep the enemy out of the camp than to expel him after he has taken possession. Here is the trouble with nine-tenths of the people in the world. They neglect precautionary measures usually through ignorance of the portent of certain symptoms, and allow diseases to become confirmed before they call a physician ; then, if they don't speedily recover, the blame is apt to fall on him. We boast of our intelligence and prosperity, and of our facilities for acquiring knowledge ; but the most important part of our education is entirely neglected.

Our Creator has given us bodies more complicated, more wonderful in their construction than the planets. Although we actually possess them, and direct and control their movements, no man is wise enough to understand how it is done. We simply know that our bodies are the ready servants of the will, which is the spirit—the immortal part. But we are not, by any means, left in ignorance of what is essential to their highest development and to their preservation. These things should be taught to our children, and the lessons repeated until they are made to understand that it is the most important knowledge they can possess. It will do more than all other influences combined to induce them to eschew evil and to lead lives consistent with the highest health, without which there can be but little happiness in life.

---

Parents who have young children should never be without "Van Deren's Remedy" for Bowel Troubles of Children. It is a certain cure in all cases.

